# Assessment of the role of emotions in audiovisual associations through an enactive approach

**DOI:** 10.1371/journal.pone.0322449

**Published:** 2025-05-23

**Authors:** Costanza Cenerini, Luca Vollero, Nicola Di Stefano, Marco Santonico, Giorgio Pennazza, Flavio Keller

**Affiliations:** 1 Department of Engineering, Unit of Electronics for Sensor Systems, Università Campus Bio-Medico di Roma, Rome, Italy; 2 Department of Engineering, Unit of Computational Systems and Bioinformatics, Università Campus Bio-Medico di Roma, Rome, Italy; 3 National Research Council, Institute of Cognitive Sciences and Technology, Rome, Italy; 4 Department of Science and Technology for Sustainable Development and One Health, Unit of Electronics for Sensor Systems, Università Campus Bio-Medico di Roma, Rome, Italy; 5 Department of Medicine, Unit of Developmental Neuroscience, Università Campus Bio-Medico di Roma, Rome, Italy; Istituto di Ricerca e di Studi in Ottica e Optometria, ITALY

## Abstract

In the expanding literature on audiovisual associations, the question of how music influences the way a person actively creates an image has received little attention. To address this gap, our study investigated the effect of music-induced emotions on several key parameters that define visual images. Following the hypothesis that creating an image while listening to emotionally evocative music would result in artwork emotionally coherent with the music, we designed a two-phase experiment. During the first phase, participants listened to ten original songs that were composed to evoke a broad range of emotions and were asked to create an image by manipulating both colour (i.e. Saturation, Brightness, Hue) and Geometric parameters (i.e., Shape, Dimension, Spatial Dispersion, and Numerosity). Participants were also required to describe the emotional responses evoked by the songs, assigning scores to a predefined set of nine musical emotions (Amazement, Solemnity, Tenderness, Nostalgia, Calmness, Power, Joyful Activation, Tension, and Sadness). In the second phase, participants viewed images with one parameter set at an extreme value while others remained at intermediate levels and were asked to describe the emotions elicited by the images. This approach enabled us to collect data on the associations between music and images, music and emotions, and images and emotions, thereby assessing whether paired audiovisual contents share the same emotional meaning. Findings reveal that sadness in music influences Brightness and Spatial Dispersion of objects in image creation. Sadness also influences Saturation. Surprisingly, amazement shared many similarities with sadness in terms of influenced parameters, namely Brightness and Dispersion, and was correlated with the colour green, typically evoking solemnity and tenderness. These findings shed novel light on the role of emotions in the mediation of audiovisual associations.

## Introduction

The term ’crossmodal associations’ is typically used to indicate the consistent matching between sensory features in one modality and features in another modality, either physically present or imagined [1]. These correspondences have been observed in normal perceivers and documented between various sensory modalities. Some correspondences have been observed to be valid across cultures and languages [2].

While crossmodal associations might evoke the phenomenon of synaesthesia, their distinction is evident. Whereas the former are consistently encountered by non-synaesthetes (and, to some degree, by synesthetes as well), the latter are idiosyncratic matchings perceived by synesthetes only [3, 4].

In research on crossmodal associations, audiovisual associations are amongst the most widely investigated correspondences, with studies mostly focusing on the association between colour and sound. Research has demonstrated that several visual features are consistently associated with auditory features, such as hue with timbre [5, 6] or pitch [7, 8], saturation with loudness and pitch [9], and brightness with pitch and loudness [10, 11]. (See [12, 13] for reviews).

In an attempt to understand the mechanisms underlying crossmodal correspondences, researchers have occasionally referred to sensory mechanisms, including associative learning, statistical co-occurrence, and at times, perceptual similarity (see [4, 14, 15]). Starting from the 1930s, many studies were conducted to test the hypothesis that emotions mediate audiovisual association, leading to the “emotional mediation hypothesis" (see [16]).

A distinction should be made between perceived and induced emotions in music psychology. Perceived emotions arise from recognizing emotional cues in music without necessarily eliciting emotional responses. Conversely, induced emotions directly evoke emotional reactions in listeners due to musical characteristics. This differentiation, outlined by Gabrielsson [17] and supported by Schubert [18], highlights significant differences, especially when the musician’s expressive intent is negative. Studies exploring the role of emotion in crossmodal associations have investigated both types.

In 1942, Odbert *et al*. [19] asked participants to associate perceived emotions and colours with a series of songs and found that songs that convey a similar emotional response were associated with similar colours, and where the participants disagreed in their emotional evaluation of the song, the colour association was inconsistent. These results were confirmed by many studies: Bresin [20] found a high correlation between the emotional intention of the performer and participants’ preference for Hue, Saturation, and Brightness: significant correlations were reported for Brightness and expressed emotions like Love, Pride, Tenderness, Contentment, Sadness, and Fear; Anger and Shame were significantly correlated with Saturation. Barbiere *et al*. [21] found that colour-music correspondences are similar among individuals, and they are correlated with perceived emotions, in such a way that sad songs are mainly associated with grey, which is correlated with Sadness, and happy songs are correlated with bright primary colours, which are correlated with positive emotions. Palmer *et al*. [1] conducted a study in which participants from the UK and Mexico were invited to choose colours that were most/least consistent with classical orchestral music by Bach, Mozart, and Brahms. The results showed that faster music in the major mode induced colour choices that were more saturated, lighter, and yellower, while slower music in minor tonality was associated with unsaturated, darker, and bluer colours. Strong correlations were found between the emotional profiles of the music and those of the colours chosen, supporting the emotional mediation hypothesis across cultures. Similar results were obtained also by Cutietta & Haggerty [22], Isbilen & Krumhansl [23], and Di Stefano *et al*. [2].

In a study involving complex stimuli, Albertazzi *et al*. [24] showcased the presence of reliable audiovisual associations between paintings and music excerpts from the classical guitar repertoire (or transcriptions, e.g., Villa-Lobos, Albeniz).

Taken together, the results from these studies suggest that emotion (either expressed or perceived) plays a fundamental role in music-colour association, thus supporting the “emotion mediation hypothesis" [1]. Based on this assumption, one might expect that, if sadness makes us see the world less bright [25, 26], listening to sad music while creating an image will likely influence the way we create the image (e.g., the created image will likely be less bright).

In the empirical literature, emotions are captured through different tools, ranging from discrete labelling, such as Ekman’s 7-emotion model [27], to a multidimensional framework like the valence-arousal model by Russell [28], as Eerola & Vuoskoski have pointed out in their review of music and emotion studies [29]. Zentner introduced the Geneva Emotional Music Scale (GEMS) in 2008, outperforming traditional representations in describing emotions from Western classical music [30]. Aljanaki [31] employs a simplified version of the GEMS which features nine emotions: Amazement, Solemnity, Tenderness, Nostalgia, Calmness, Power, Joyful Activation, Tension, and Sadness.

In the existing literature, most studies passively involved subjects by exposing them to visual and musical stimuli and offering a predetermined set of alternatives from which to choose. However, based on an enactive approach to perception and cognition, according to which organisms gain knowledge of the world through dynamic interactions with their environment (see, e.g., [32]) and to an embodied view of human intelligence [33], humans should be actively involved in the experimental paradigm. Furthermore, none of the studies discussed above conducted an in-depth analysis of the role of Shape and Spatial Distribution of the image in the association, focusing only on colour dimensions such as Hue, Saturation, and Brightness (though see [34] for a possible exception).

To fill this gap, we developed an experimental protocol to investigate how perceived musical emotions influence image creation. Participants were invited to actively create novel images by acting on several parameters, such as Colour, Shapes, and Spatial Distribution of graphic elements upon hearing a song, and they were then asked to describe the emotions elicited by listening using the GEMS reduction by Aljanaki [31]; later they were shown some images and asked to describe again their emotions. The results substantiate the role of emotions in the crossmodal association between music and colour, emphasizing its effects on perceiving Shapes and Spatial Organization of elements in images. Specifically, the results showed that music associated with Amazement influenced the Shape of generated objects, Sadness impacted the Spatial Dispersion of the objects, and Calmness influenced the Number of objects.

## Materials and methods

This study received the approval of Università Campus Bio-Medico di Roma’s ethical committee on February 16, 2022, with number of clinical studies’ register 2021.236. We affirm that all methods employed in this study were conducted in strict adherence to the applicable guidelines and regulations.

The protocol employed in this study underwent initial testing in a pilot trial with the beta version administered to 15 subjects. Early findings and subsequent adjustments, informed by feedback from this trial, were presented at the ACAIN conference in September 2022 [35]. In this paper, we present the results of the modified protocol developed post the pilot trial phase, involving 80 participants.

The test was conducted remotely through a website hosted on Digital Ocean (https://www.digitalocean.com/). Results were securely stored in a proprietary database hosted on Firebase (https://firebase.google.com/), a service provided by Google.

### Participants

Over a span of 2 months, from 2nd April to 2nd June 2022, Italian-speaking participants were recruited for the study. The recruitment was based on a predetermined time frame rather than targeting a specific sample size, with the final number reflecting all eligible volunteers who joined during this period. An a priori power analysis indicated that for detecting large effects (f = 0.4) in our planned analyses, approximately 55 participants would be needed. Prior to their involvement in the trial, individuals were required to complete and sign the informed consent form as well as the privacy policy. Enrolment took place in two parts: in the first part, in the month of April, 84 subjects were recruited and then split into two groups, namely Group A and Group B, both comprising 42 subjects; in the second part, in the month of May, 39 subjects were recruited to be added to Group A (which thus increased up to a final number of 81 subjects). The total number of participants was 123 (M=56 (45.53%), F=67 (54.47%), mean age=23.4, SD=4.7). Exclusion criteria included colour blindness and incorrect completion of the key phases of the test. Out of this group, 39 participants did not start the test after the enrolment, and 4 started the test but did not finish it, therefore the final number of subjects analysed was 80 (M=34 (42.5%), F=46 (57.5%), mean age=23.07, SD=5.3). During the experiment, 80% of participants chose to complete the test in one session, while the remaining participants opted to finish it in multiple sessions. For those who chose multiple sessions, no interval exceeded one week between sessions. Group A was composed of 47 subjects (M=22 (46.8%), F=25 (53.2%), mean age=22.34), Group B of 33 subjects (M=15 (45.5%), F=18 (54.5%), mean age=24.12).

Participants were required to fill out a form that gathered demographic information as well as details on musical and artistic background and ratings, emotional responses to specific colours and shapes, and any previous experiences with synaesthesia. Upon receiving the experiment link via email, participants could complete the test in one or multiple sessions.

### Test protocol

#### Pretest.

The pretest comprises five distinct evaluations designed to assess the visual and musical capabilities of the subjects. The tests included are:

- Ishihara Test [36]: this test is a diagnostic examination to assess an individual’s ability to perceive colours. Comprising plates of coloured dots, the test is designed to detect the presence of colour blindness and determine the specific type of colour vision deficiency an individual may have.
- Perfect Pitch Test: “Perfect pitch," or absolute pitch, is the ability to recognize and identify a musical note immediately without the need for a reference. In the context of an absolute pitch test, individuals are presented with a series of musical notes and asked to identify each note without external assistance. This test aims to assess the accuracy and consistency with which a person can recognize specific sound frequencies, thereby providing a measure of absolute pitch ability.
- Melodic Discrimination Test (adapted from Harrison *et al*. [37]).
- Mistuning Perception Test (adapted from Larrouy *et al*. [38]).
- Beat Alignment Test (adapted from Harrison *et al*. [39]).

The adaptation made to the last three tests was that, in this case, we presented a subset of 5 stimuli in a random order, instead of the whole set of stimuli in an increasing difficulty order.

#### First phase.

This phase of the study aims to assess the music-induced emotions experienced by the subjects and their ability to generate mental imagery from music. During this phase, participants listened to a specific song while simultaneously viewing an image on the screen. They had the opportunity to modify the image by adjusting 7 sliders that control the colour of the objects, represented using the Munsell colour space [40], a standardized system that delineates Hues, Saturation, and Brightness (HSB) parameters, and graphical parameters of the objects in the image: Number, Dimension, Dispersion, and Shape. They were asked to listen to the music and modify the image they were seeing so that it would match the one forming in their mind.

Subsequently, participants were able to listen to the same song again and were asked to describe their emotional response using a 9-point emotional scale, which represents a Reduction of the GEMS emotion model [31]. This process was repeated for a total of 10 songs. The songs, composed by a professional musician specifically for the study, represented different musical genres, each lasting approximately 1.30 minutes, with a range from 78 to 106 seconds. They were artificially synthesized using the music software Logic Pro X. The files created were of MIDI type, except for the piano performances and some of the percussion, which were recorded in audio format. The singer’s voice is a sample available online. An overview of the features of the songs can be found in Table 1.

**Table 1:** Information about musical tracks presented in the first phase of the test.

Track Number	Genre	Key	BPM
1	Choir	Fmin	140
2	POP	Dmaj	140
3	Orchestral Soundtrack	Bmin	94
4	Trap	Cmin	110
5	Electronic	Cmin	128
6	Minimal Soundtrack	Emaj	90
7	Country	Gmaj	74
8	Jazz	Dmaj	90
9	Classical	Bmaj	100
10	Latino	Amin	90

#### Second phase.

**Fig 1:**
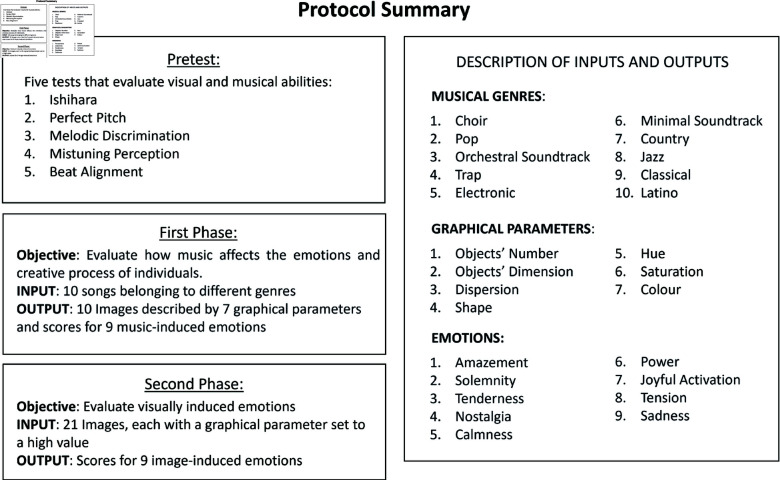
Schematic representation of the test phases. The test was administered remotely, and subjects could complete the pretest and the two main phases in a single or in separate sessions. Subjects belonging to Group A completed all the phases, whereas subjects from Group B skipped phase one.

In this phase of the study, participants were presented with 21 images that had been generated using the same parameters that participants were able to modify in the first phase. Each image had neutral values for most parameters, except for one specific parameter that was intentionally exaggerated (e.g., an image with very high Brightness). Upon viewing each image, participants were once again prompted to describe their emotional experience using the same 9 emotions as in the first phase. A summary of the variables involved in the test can be visualized in Fig 1.

### Familiarity bias assessment

Familiarity bias can be described as the tendency to seek confirmation of expectations, retaining, or avoiding abandoning favoured hypotheses or choices [41]. During the beta testing of the protocol, some subjects reported that they felt inclined to align their choices in the second phase with their selections in the first phase instead of answering spontaneously, potentially introducing a familiarity bias in the results. To address this concern, a decision was made to divide the subjects into two groups: Group A, which completed the entire protocol, and Group B, which only completed the pretest and the second phase.

The purpose of this division was to establish a standard reference for a typical emotional response to the images by observing Group B’s results. Subsequently, the responses of Group A could be compared to this reference to determine if there were any deviations to be addressed to the completion of phase one, thereby assessing the presence of a familiarity bias.

#### Division in Groups A and B.

**Fig 2:**
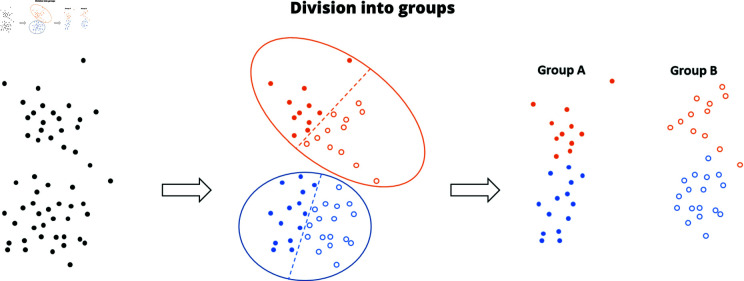
Process of division into groups A and B. The dots in the figure represent individual subjects positioned in the feature space, which has been simplified to 2D for clarity. Process of division into Group A and Group B: We applied the k-means algorithm to the responses provided in the form (black dots) and identified two significantly different groups (yellow vs blue dots), indicating a stratification of the subjects. Consequently, we split (dotted lines) and recombined these groups to obtain Group A and Group B, which exhibit a similar distribution.

To ensure an equal distribution among the groups, participants were divided based on their responses to the enrolment form. Firstly, the k-means clustering algorithm was applied to the form results to verify the absence of any hidden stratifications [42] in the subject population. This highlighted the presence of two significantly different groups according to the results of the Kruskal-Wallis test (p < 0.001). Then, Group A and Group B were formed by selecting equal random proportions from the two clusters (see Fig 2). The Kruskal-Wallis test was applied to compare these new two groups (Group A and Group B), and the results showed no significant difference (p > 0.05).

### Analysis of the relevance of the emotion in the whole dataset

The final analysis focuses on all subjects from Group A, aiming to assess the emotional correspondence between the emotions induced by music during the image generation process and the emotions evoked by viewing the images in phase two. To achieve this, we computed the mean of each graphical parameter for each subject. Subsequently, for each emotion and graphical parameter, the following steps were undertaken:

1. Identify the songs from phase one where the graphical parameter of the corresponding generated image is higher than the mean.
2. Compare the similarity of the emotions indicated for the track corresponding to the image generated in the first phase and the corresponding one from the second phase and average over each emotion:

$$\text{emotional consistency}=1-\frac{\left|A(i)-B(i)\right|}{100}$$ (1)

in which: *i = emotion, A = emotional spectra from phase 1, B = emotional spectra from phase 2*

The mean per subject and the standard deviation of the emotional consistency were then computed.

A low value of emotional correspondence indicates a disagreement between the music-induced emotions of the first phase and those induced by the sight of images with high parameters in the second phase. In such cases, the specific emotion was not considered relevant in the association between the music heard and the generated image. Conversely, a high value suggests that the emotions expressed in the two phases were similar. Some examples of possible values are reported in Table 2.

**Table 2:** Example of possible values given in the two phases and their relative emotional correspondence index.

Emotion in Phase 1	Graphical parameter in Phase 1	Emotion in Phase 2	Emotional correspondence index
100	70	100	1
0	10	0	1
50	100	50	1
0	20	80	0.2
20	80	70	0.5

After computing the emotional correspondence, the authors applied different techniques to find clusters of subjects with different behaviours but could not find any groups with significant differences.

### Insights on music-induced emotions

To gain a deeper understanding of the emotions induced by music, we calculated Kendall’s correlation coefficient between the features of the songs and the emotions expressed in phase one. Utilizing the MIRToolbox on MATLAB R2020a, 12 loudness, timbral, rhythmic, and tonal features were extracted from each song. None of these correlation values exceeded 0.5.

We extended this analysis by computing the same parameter for the emotions in phase one and the results from the pretest, aiming to assess how one’s musical abilities influence the perception of music-induced emotions. In this case as well, the correlation values were lower than 0.5, indicating that musical abilities do not significantly impact the perception of emotions.

For additional details on music-induced emotions, please refer to page 1 of the supporting information.

#### Groups A and B comparison.

Once the subjects completed the experiment, the results from Groups A and B were analysed to investigate the potential influence of phase one on phase two. Two main comparisons were conducted.

The first comparison focused on the emotional response to the neutral image, which served as the baseline for analysis. The second comparison involved all the other images. For each subject, the emotions evoked by the neutral image were subtracted from the emotions evoked by the other images. This approach allowed for the examination of emotional variations rather than absolute emotional states.

Subsequently, the distribution of each image was compared between the experimental and control groups using the Kruskal-Wallis test.

In addition to the Kruskal-Wallis test, we calculated the effect size using eta squared ($\eta^{2}$) to quantify the magnitude of the differences between groups. Eta squared was computed from the chi-square statistic using the formula $\eta^{2}$ = chi-square/(n-1), where n is the total number of observations. To interpret the magnitude of the effect sizes, we followed conventional guidelines: $\eta^{2}\approx$ 0.01 indicates a small effect, $\eta^{2}\approx$ 0.06 indicates a medium effect, and $\eta^{2}\approx$ 0.14 indicates a large effect [43].

After confirming the absence of familiarity bias, Group B was excluded from further analysis, and only data from Group A were considered for subsequent analysis.

## Results

In order to assess the consistency of the observed associations within subjects, we devised an experimental paradigm consisting of two phases: in Phase 1, participants were allowed to actively modify all parameters of an image while listening to musical excerpts and then rate 9 emotions to describe the feeling induced by the song. In Phase 2, they were shown a “neutral" image and a series of images where all image parameters, except one, were set at an average level. They were then asked again to rate the same 9 emotions. Participants were divided into two groups. Group A completed the whole test, while Group B completed only Phase 2. This was necessary to test whether a familiarity bias emerges between the two phases. Analysis of the data from the second phase of both groups indicated there was no statistically significant difference between them, thus making familiarity bias in the test unlikely.

### Robustness and impartiality of the results

**Fig 3:**
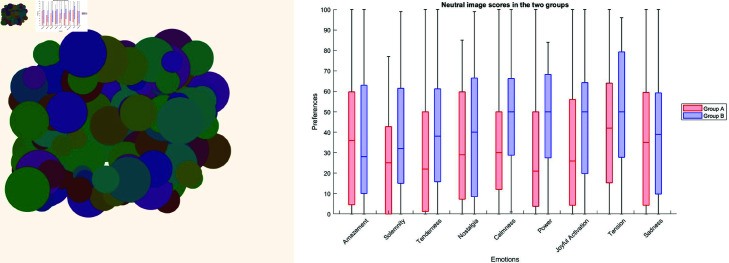
Initial emotional response differences between groups. (A) The neutral image shown to Group A and B as a reference for the emotional state at the beginning of phase 2. The neutral image was generated with parameters including saturation, brightness, dimension, dispersion, shape, and numerosity were set at the median value within its respective range, except for hue, which varies for each object contained in the image, covering the entire 360${\;}^{\circ }$ range of the colour wheel. (B) Boxplot illustrating the distinct emotional reactions between participants from Group A, who completed the first phase before viewing this neutral image, and participants from Group B, who did not undergo phase one. The scale of ratings in the boxplot ranges from 0 to 100, where 100 represents a strong preference and 0 represents a low preference. This finding suggests that the completion of phase one has modified the emotional baseline of Group A.

A comparison between the emotional spectra induced by the neutral image in both groups is shown in Fig 3. The application of the Kruskal-Wallis test to these data revealed a significant difference between the two groups (*p* < 0.01). This indicates that completion of Phase 1 resulted in an alteration of the emotional state. Emotions such as Tenderness, Calmness, Power, and Joyful Activation were particularly affected, as illustrated in the boxplot in Fig 3.

Conversely, the effect of the first phase on the variations of emotions triggered by all the other images did not reach significance (*p* > 0.05), indicating that there is no familiarity bias in the test, but just a modification of the emotional baseline that does not affect individual associations.

**Fig 4:**
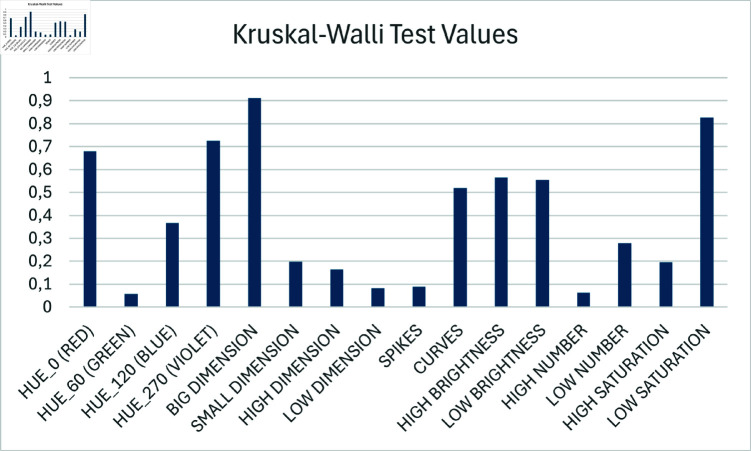
Emotional response comparison between groups. The Kruskal-Wallis test was employed to examine differences in the emotional response between Group A and Group B when exposed to the images during phase 2. In this phase, subjects were shown images where all image parameters were set at a median value, except one parameter that was set at an extreme value. The emotional responses were measured as differences from each subject’s response to the neutral image. Bars represent p-values. The analysis revealed that there was not a statistically significant difference between the two groups (*p* > 0.05).

The calculated effect size for the comparison of emotional responses to the neutral image was substantial ($\eta^{2}$ = 0.2439), indicating a large effect according to conventional guidelines and suggesting that completing Phase 1 had a considerable impact on participants’ baseline emotional state.

Conversely, when analysing the normalized emotional responses (variations from neutral image) for all other images, the effect sizes were notably smaller, as detailed in Table 3. Most parameters showed small or negligible effect sizes ($\eta^{2}<0.06$), with only “Red" ($\eta^{2}=0.0773$), “Low Dispersion" ($\eta^{2}=0.0903$), and “High/Low Number" ($\eta^{2}\approx 0.062$) reaching medium effect sizes. These substantially reduced effect sizes for normalized responses confirm that while Phase 1 altered the emotional baseline, it did not affect the individual patterns of audiovisual associations, thus supporting the absence of a familiarity bias in our experimental design.

**Table 3:** Effect sizes ($\eta^{2}$) for comparisons between Group A and Group B. Effect sizes are color-coded according to magnitude: yellow ($\eta^{2}<0.01$) indicates negligible effect, orange ($0.01\leq \eta^{2}<0.06$) indicates small effect, red ($0.06\leq \eta^{2}<0.14$) indicates medium effect, and red ($\eta^{2}\geq 0.14$) indicates large effect.

Comparison	$\eta^{2}$
Emotional response to neutral image	0.2439
**Normalized emotional responses by image parameter**	
Green	0.0148
Red	0.0773
Light Blue	0.0001
Purple	0.0001
Big Dimension	0.0010
Small Dimension	0.0338
High Dispersion	0.0223
Low Dispersion	0.0903
Spikes	0.0464
Curves	0.0225
High Brightness	0.0163
Low Brightness	0.0205
High Number	0.0623
Low Number	0.0621
High Saturation	0.0392
Low Saturation	0.0125

### The influence of music-induced emotions in image creation

To quantify the influence of emotion on audiovisual association, we computed an “emotional correspondence index" between the emotions expressed in the first and second phase by each subject. This coefficient compares the music-induced emotions that lead to creating images with a high graphical parameter value in the first phase (e.g., Colour Saturation) to the emotions expressed upon seeing the image with the same set of parameters in the second phase (i.e., Colour Saturation is set at high value, and all other parameters are set at a neutral value). Theoretically, the emotional correspondence index can vary from 0 (no correspondence) to 1 (perfect correspondence). A high emotional correspondence index indicates that the ratings given to a given emotion in the two phases are similar, suggesting that the emotion had a relevant role in the association, whereas a low value indicates that they are dissimilar, and thus the emotion did not influence the association.

The results are shown in Table 4.

**Table 4:** Emotional correspondence index.

Image parameters	Solemnity	Tension	Power	Amazement	Sadness	Nostalgia	Tenderness	Calmness	Joyful Activation
Green	0.71	0.67	0.67	0.72	0.66	0.68	0.75	0.68	0.62
Red	0.68	0.62	0.65	0.66	0.68	0.71	0.67	0.61	0.63
Light Blue	0.64	0.67	0.65	0.64	0.72	0.70	0.69	0.64	0.64
Purple	0.65	0.70	0.67	0.66	0.64	0.66	0.70	0.69	0.60
High Brightness	0.68	0.72	0.67	0.73	0.76	0.70	0.66	0.68	0.67
Low Brightness	0.73	0.74	0.73	0.69	0.76	0.67	0.65	0.69	0.62
High Saturation	0.71	0.71	0.70	0.68	0.70	0.67	0.70	0.72	0.62
Low Saturation	0.71	0.67	0.70	0.73	0.75	0.71	0.71	0.67	0.65
Big Dimension	0.62	0.68	0.67	0.67	0.67	0.71	0.71	0.67	0.64
Small Dimension	0.64	0.63	0.63	0.68	0.74	0.72	0.69	0.63	0.65
Spikes	0.68	0.63	0.66	0.70	0.71	0.70	0.67	0.64	0.58
Curves	0.67	0.61	0.69	0.75	0.74	0.73	0.73	0.66	0.67
High Number	0.63	0.53	0.67	0.62	0.50	0.71	0.67	0.61	0.50
Low Number	0.70	0.67	0.71	0.70	0.70	0.66	0.71	0.68	0.68
High Dispersion	0.67	0.69	0.64	0.69	0.71	0.70	0.68	0.68	0.65
Low Dispersion	0.72	0.72	0.70	0.74	0.78	0.68	0.72	0.66	0.70

Sadness is the emotion with the highest emotional correspondence indexes: it is perceived similarly in the first and second phase when dealing with images with low Dispersion (0.78), high or low Brightness (0.76 in both cases), and low Saturation (0.75). Second to Sadness, Amazement showed a high emotional consistency in the perception of Curvilinear shapes (0.75). Many other emotion-graphical parameter relationships are worth mentioning:

- Sadness influences almost every graphical parameter, except for Hue, Big Dimension, and high Number.
- Amazement influences both Curvilinear and Spiked shapes, and parameters like low Dispersion and low Saturation.
- Tenderness influences many parameters, with a high score for Green (0.75). The most dichotomic image parameter, i.e. the one that showed the most difference between a low and a high value, was Numerosity: low Numerosity was associated with Tension, Sadness, and Joyful Activation.
- Nostalgia influences both Spiked and Curvilinear shapes (0.70 and 0.73) and Dimensions (0.71 and 0.72).
- Power and Solemnity are both relevant in both directions of Saturation (0.70 in both directions for Power and 0.71 in both directions for Solemnity) and low Brightness (respectively, 0.73 and 0.74).
- Tension influences the perception of Brightness (0.72 and 0.74).

## Discussion

This paper presents the results of an innovative music-emotion-image association test conducted with 80 participants. The novelties of this study are the enactive experimental approach, which actively involved participants in the generation of visual images, and the parameters investigated, which included geometrical properties such as Shape, Dimension, Numerosity, and Spatial Dispersion. The experimental design guarantees that the emerging relationships between the three variables (music, emotions, and image parameters) are likely due to spontaneous cross-modal interactions, as completing Phase 1 does not affect the answers given in Phase 2.

The analysis of effect sizes (Table 3) provides additional insight into our methodological approach. While the Kruskal-Wallis test showed a significant difference between groups in baseline emotional responses to the neutral image (*p* < 0.01), the large effect size ($\eta^{2}$ = 0.2439) quantifies the magnitude of this difference, confirming that completing Phase 1 substantially altered participants’ emotional state. Crucially, when examining normalized responses across different parameters, not only did the Kruskal-Wallis test show no significant diffe-rences (*p* > 0.05), but the effect sizes were also predominantly small. This combination of non-significant test results and small effect sizes provides robust evidence that the baseline emotional shift did not influence the patterns of audiovisual associations. These quantitative findings validate our experimental design and support our conclusion that the observed relationships between music, emotions, and image parameters reflect genuine cross-modal interactions rather than methodological artifacts.

Before delving into the results, it’s crucial to emphasize the significance of the values presented in Table 4. These values indicate the degree of consistency exhibited by participants in expressing emotions during both Phase 1 and Phase 2. Specifically, they illustrate the extent to which the emotions elicited by the music during Phase 1, which led to the creation of an image, closely aligned with those expressed during Phase 2. Essentially, we assessed how the music influenced the emotional responses that guided image creation, without considering the directionality of this influence. Therefore, it’s possible for an emotion to be associated with two opposite ranges of the same parameter (e.g., Sadness with both High and Low Brightness), indicating that Sadness influences Brightness across its entire range.

The results presented in Table 4 align with existing literature on the interplay between emotion and the association between music and colour. Previous studies, such as Barbiere *et al*. [21], have reported correlations between specific colours and emotional states induced by music. For instance, grey has been linked to sadness [21], which is in line with our findings indicating that high and low Brightness are both influenced by Sadness. Similarly, bright colours have been associated with happiness [21], which is supported by our observation of a high emotional correspondence index between high Brightness and emotions such as Amazement, albeit also with Sadness. Furthermore, green has been correlated with happiness [21], consistent with its elevated emotional correspondence values with emotions like Amazement and Tenderness in our study.

The findings not only confirm existing knowledge regarding the role of emotions in crossmodal connections between music and colour but also yield novel insights into their impact on associations with graphical parameters such as Shape, Dimension, Numerosity, and Dispersion. To our knowledge, this relationship between emotions and geometrical parameters in the context of image creation has never been analysed before in detail. Studies suggest that the geometrical characteristics of spaces and environments can influence emotional states. For example, people tend to prefer objects with curvilinear contours rather than sharp contours [44]. Also, the layout and geometry of a room or building may contribute to feelings of comfort, arousal, or relaxation (see [45]). Our study indicates that this relationship is reciprocal, reinforcing the relevance of the association.

Several factors should be considered when interpreting the findings of this study. The experimental sample consisted of Italian participants aged between 18 and 40. While this homogeneous sample may limit generalizability, it also provides insights into a specific demographic. Future studies could benefit from including participants of diverse ages and nationalities to enhance generalizability.

Regarding the musical stimuli, they successfully elicited a wide range of emotions (see Supporting Information), and using original compositions minimized the influence of prior associations on emotional responses. The number and duration of stimuli ensured a good balance between study length and the breadth of emotions elicited, considering the potential for participant fatigue—a significant concern in emotion measurement studies—over extended testing periods. Despite this, exploring a wider variety of musical sources could enrich the understanding of emotional responses.

Our findings are relevant to many areas of artistic activity, especially for art forms that make ample use of combinations of visual effects and music to evoke specific emotions in the viewer, such as films, operas, etc. For example, our results show that musical Sadness influences geometrical properties like low Numerosity, low Dispersion, and colour properties like low Saturation and light blue Colour. Such empirical evidence could become the basis for fruitful interactions between researchers and artists like painters, filmmakers, and opera directors. This could provide empirical evidence to common practices in the field of artistic audiovisual creation, such as film or opera. Additionally, these data could be used to try to predict some general features of the image that a viewer may associate with a song based on prior knowledge of the emotions evoked by listening to music. In this way, an expert system could be trained to reproduce audio-visual associations in healthy subjects and then used to “play" images or “show" songs to people with sensory impairments, allowing the development of new tools to help them perceive the emotional content of the affected sense. This extension of the present work will require more extensive data collection with the already tested protocol and platform presented in this paper.

## Supporting information

S1 Fig Mean and standard deviation of emotional scores across songs. Mean and standard deviation of the scores of each emotion for each of the songs. Each score ranges from 0 (center of the plot) to 100 (extremities of the plot), with lines at 33.3 and 66.6, representing intermediate points.
(TIF)

S2 Fig Correlation between musical features and emotions. Correlation coefficient between musical features and emotion induced by hearing the songs in phase one. The musical features are extracted using the MIRToolbox in MATLAB.
(TIF)

S3 Fig Correlation between pretest scores and emotions. Correlation coefficient between pretest scores and emotion induced by hearing the songs in phase one. MPT = Mistuning Perception Test, aiming at evaluating the ability to discerning tune and out of tune songs, BAT = Beat Alignment Test, that evaluates the ability of recognizing on time and out of time percussion, and MDT = Melodic Discrimination Test, that evaluates the ability to discern different melodies.
(TIF)

S1 File Original questionnaire in Italian. Complete questionnaire administered to participants, containing all questions about demographic information, musical/artisticbackground, personal preferences, emotional responses to specific colours and shapes, and prior experiences with synaesthesia.
(PDF)

S2 File English translation of the questionnaire. English translation of the complete questionnaire, providing the same questions as in S1 File for international readers.
(PDF)

S1 Data Musical stimuli. The songs utilized as stimuli in the protocol can be accessed at: https://drive.google.com/drive/folders/1li5TKqhGgFZxzkdYUdriveink.
(PDF)
