# Supplementary figures and images for: Assessment of the role of emotions in audiovisual associations through an enactive approach
Source: PLoS One. 2025 May 23;20(5):e0322449. doi: 10.1371/journal.pone.0322449 (PMC12101654; doi:10.1371/journal.pone.0322449)

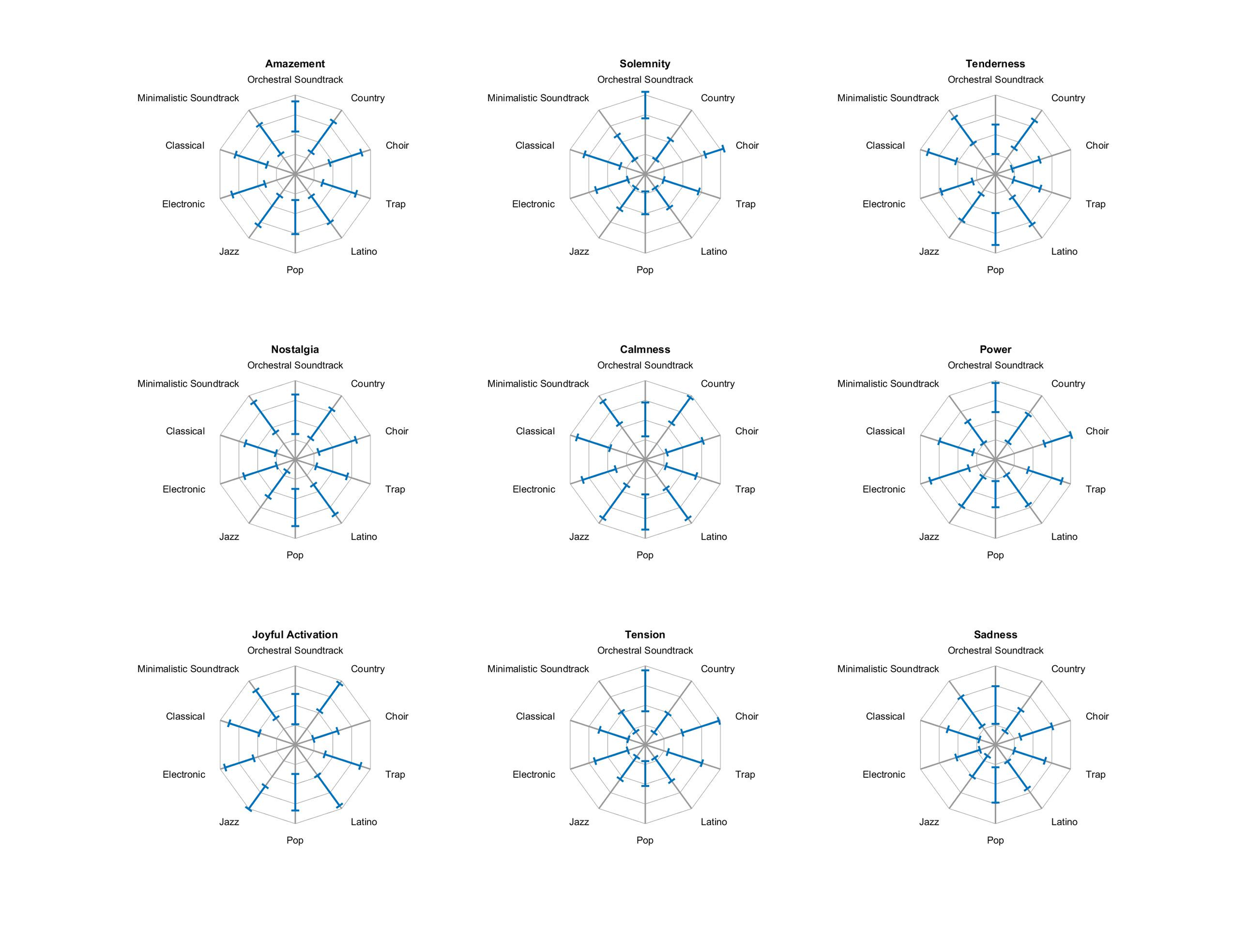

Supplement: S1 Fig — Mean and standard deviation of the scores of each emotion for each of the songs. Each score ranges from 0 (center of the plot) to 100 (extremities of the plot), with lines at 33.3 and 66.6, representing intermediate points. (TIF) [file pone.0322449.s001.tif]

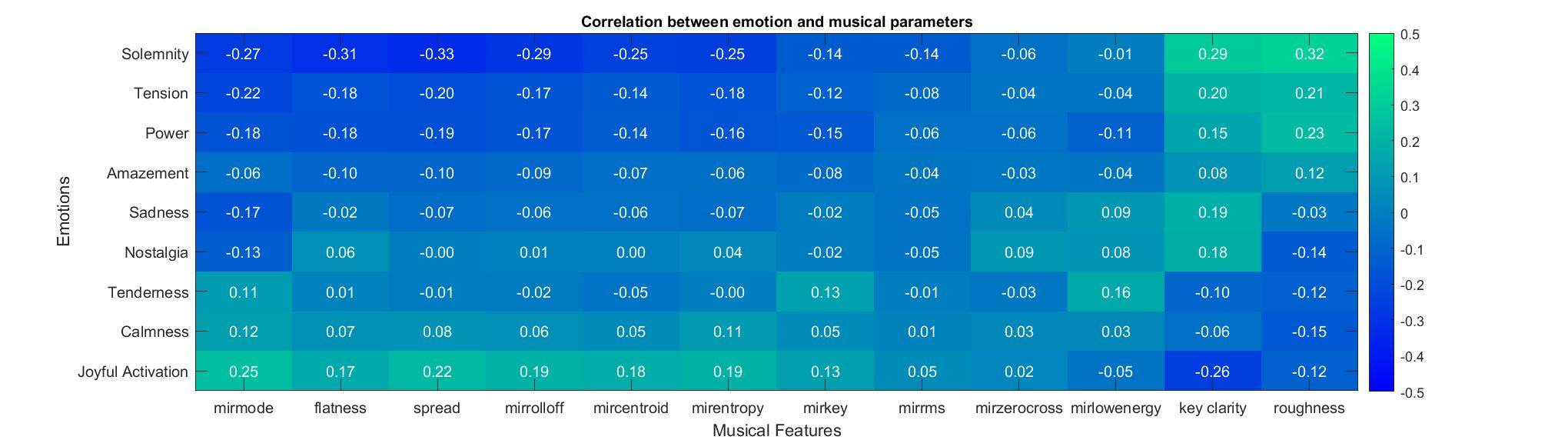

Supplement: S2 Fig — Correlation coefficient between musical features and emotion induced by hearing the songs in phase one. The musical features are extracted using the MIRToolbox in MATLAB. (TIF) [file pone.0322449.s002.tif]

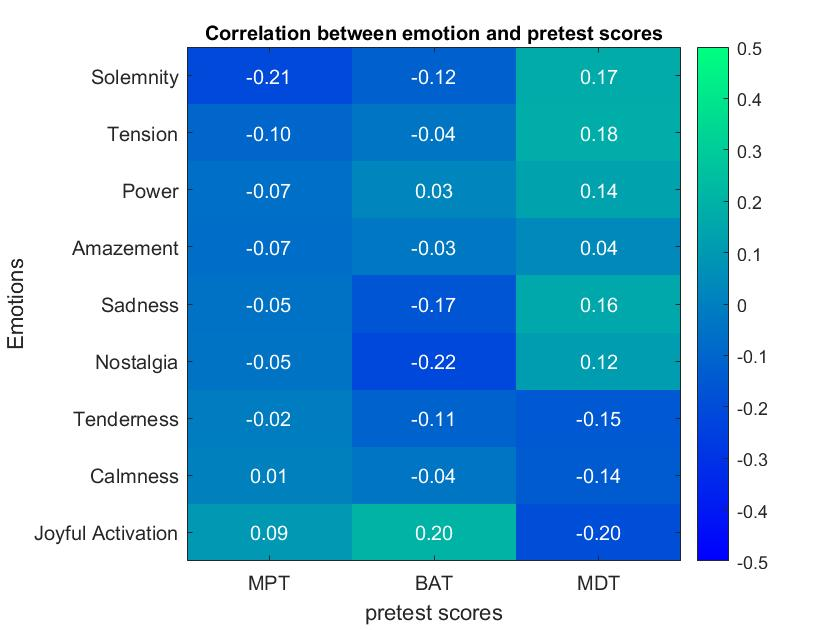

Supplement: S3 Fig — Correlation coefficient between pretest scores and emotion induced by hearing the songs in phase one. MPT = Mistuning Perception Test, aiming at evaluating the ability to discerning tune and out of tune songs, BAT = Beat Alignment Test, that evaluates the ability of recognizing on time and out of time percussion, and MDT = Melodic Discrimination Test, that evaluates the ability to discern different melodies. (TIF) [file pone.0322449.s003.tif]
