# Supplementary material for: Assessment of the role of emotions in audiovisual associations through an enactive approach
Source: PLoS One. 2025 May 23;20(5):e0322449. doi: 10.1371/journal.pone.0322449 (PMC12101654; doi:10.1371/journal.pone.0322449)
Supplement: S1 File — Complete questionnaire administered to participants, containing all questions about demographic information, musical/artisticbackground, personal preferences, emotional responses to specific colours and shapes, and prior experiences with synaesthesia. (PDF) [file pone.0322449.s004.pdf]

Informazioni di base

Rispondi a queste domande prima di iniziare il test.

* Indica una domanda obbligatoria

1. Email *

2. Nome e Cognome

3. Data di nascita *

   Esempio: 7 gennaio 2019

4. Sesso *

   Contrassegna solo un ovale.

   - Uomo
   - Donna
   - Preferisco non rispondere

5. Nazionalità *

6. Con che mano utilizzi il mouse? *

   Contrassegna solo un ovale.

   - Destra
   - Sinistra
   - Non uso sempre la stessa mano

Background musicale

7. In media, quanto tempo ascolti brani quotidianamente? *

Contrassegna solo un ovale.

- Meno di 20 minuti
- 20 minuti - 1 ora
- 1-3 ore
- Più di 3 ore

Indica su una scala da 1 a 10 il tuo gradimento nei confronti dei diversi generi musicali:

8. Classico *

Contrassegna solo un ovale.

1	2	3	4	5	6	7	8	9	10

9. Pop *

Contrassegna solo un ovale.

1	2	3	4	5	6	7	8	9	10

10. Rock *

Contrassegna solo un ovale.

1	2	3	4	5	6	7	8	9	10

11. Jazz e Blues *

Contrassegna solo un ovale.

1	2	3	4	5	6	7	8	9	10

12. Hip hop e rap *

Contrassegna solo un ovale.

	1	2	3	4	5	6	7	8	9	10	
	◯	◯	◯	◯	◯	◯	◯	◯	◯	◯	

13. Country-Western *

Contrassegna solo un ovale.

	1	2	3	4	5	6	7	8	9	10	
	◯	◯	◯	◯	◯	◯	◯	◯	◯	◯	

14. Elettronica *

Contrassegna solo un ovale.

	1	2	3	4	5	6	7	8	9	10	
	◯	◯	◯	◯	◯	◯	◯	◯	◯	◯	

15. Colonne sonore *

Contrassegna solo un ovale.

	1	2	3	4	5	6	7	8	9	10	
	◯	◯	◯	◯	◯	◯	◯	◯	◯	◯	

16. Hai mai partecipato a delle lezioni di musica? *

Contrassegna solo un ovale.

◯ Sì *Passa alla domanda 17.*

◯ No *Passa alla domanda 20.*

Background musicale

17. Per quanti anni hai studiato musica? *

Contrassegna solo un ovale.

	0	1	2	3	4	5	6	7	8	9	10	
Men	◯	◯	◯	◯	◯	◯	◯	◯	◯	◯	◯	Per 10 o più anni

18. Quale/i strumento/i hai studiato? *

____

19. Quando hai partecipato all'ultima lezione? *

Contrassegna solo un ovale.

- ◯ Meno di 1 anno fa
- ◯ Meno di 2 anni fa
- ◯ Meno di 5 anni fa
- ◯ Meno di 10 anni fa
- ◯ Più di 10 anni fa

Background artistico

In una scala da 1 a 10, indica quanto trovi emozionanti questi colori e forme:

20. Rosso *

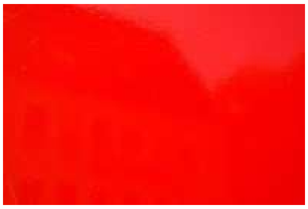

Contrassegna solo un ovale.

1	2	3	4	5	6	7	8	9	10
◯	◯	◯	◯	◯	◯	◯	◯	◯	◯

21. Giallo *

Contrassegna solo un ovale.

	1	2	3	4	5	6	7	8	9	10	

22. Blu *

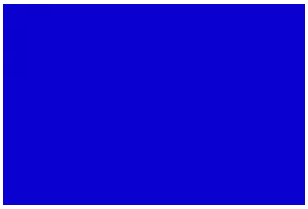

Contrassegna solo un ovale.

	1	2	3	4	5	6	7	8	9	10	

23. Arancione *

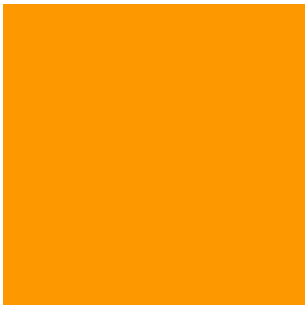

Contrassegna solo un ovale.

	1	2	3	4	5	6	7	8	9	10	

24. Verde *

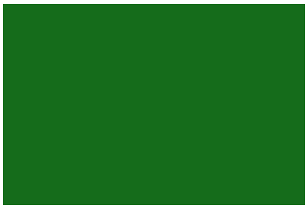

Contrassegna solo un ovale.

	1	2	3	4	5	6	7	8	9	10	
	◯	◯	◯	◯	◯	◯	◯	◯	◯	◯	

25. Viola *

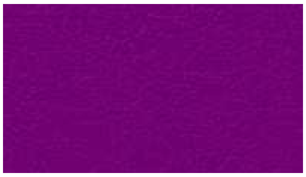

Contrassegna solo un ovale.

	1	2	3	4	5	6	7	8	9	10	
	◯	◯	◯	◯	◯	◯	◯	◯	◯	◯	

26. Bianco *

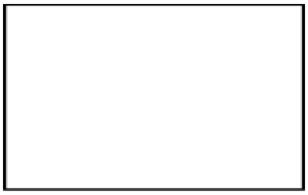

Contrassegna solo un ovale.

	1	2	3	4	5	6	7	8	9	10	
	◯	◯	◯	◯	◯	◯	◯	◯	◯	◯	

27. Nero *

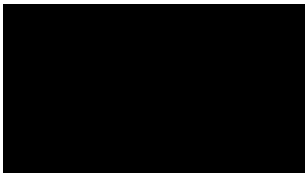

Contrassegna solo un ovale.

	1	2	3	4	5	6	7	8	9	10	
	◯	◯	◯	◯	◯	◯	◯	◯	◯	◯	

28. Grigio *

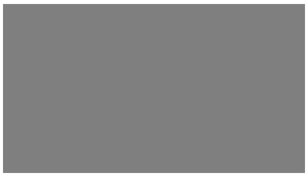

Contrassegna solo un ovale.

	1	2	3	4	5	6	7	8	9	10	
	◯	◯	◯	◯	◯	◯	◯	◯	◯	◯	

29. Esagono *

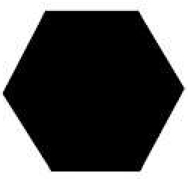

Contrassegna solo un ovale.

	1	2	3	4	5	6	7	8	9	10	
	◯	◯	◯	◯	◯	◯	◯	◯	◯	◯	

30. Triangolo *

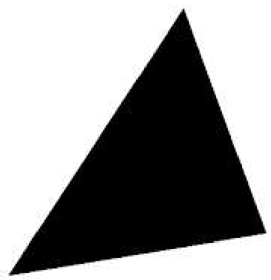

Contrassegna solo un ovale.

1	2	3	4	5	6	7	8	9	10
◯	◯	◯	◯	◯	◯	◯	◯	◯	◯

31. Quadrato *

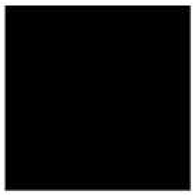

Contrassegna solo un ovale.

1	2	3	4	5	6	7	8	9	10
◯	◯	◯	◯	◯	◯	◯	◯	◯	◯

32. Ellisse *

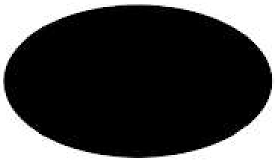

Contrassegna solo un ovale.

1	2	3	4	5	6	7	8	9	10
◯	◯	◯	◯	◯	◯	◯	◯	◯	◯

33. Cerchio *

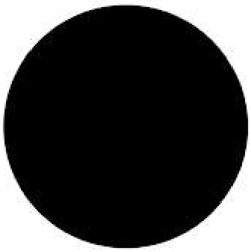

Contrassegna solo un ovale.

1	2	3	4	5	6	7	8	9	10
◯	◯	◯	◯	◯	◯	◯	◯	◯	◯

34. Splash *

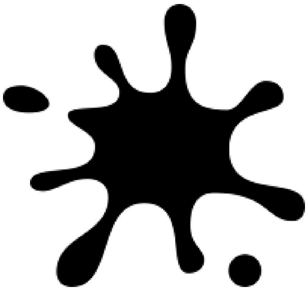

Contrassegna solo un ovale.

1	2	3	4	5	6	7	8	9	10
◯	◯	◯	◯	◯	◯	◯	◯	◯	◯

35. Hai mai partecipato a lezioni di storia dell'arte, pittura, disegno, scultura o simili? *

Contrassegna solo un ovale.

- Sì *Passa alla domanda 36.*
- No *Passa alla domanda 39.*

Background artistico

36. A che genere di lezioni hai partecipato? *

____

37. Per quanti anni? *

Contrassegna solo un ovale.

	0	1	2	3	4	5	6	7	8	9	10	
Men												Da 10 o più anni

38. Quando hai partecipato all'ultima lezione? *

Contrassegna solo un ovale.

- Meno di 1 anno fa
- Meno di 2 anni fa
- Meno di 5 anni fa
- Meno di 10 anni fa
- Più di 10 anni fa

Background medico

39. Ti sono mai stati diagnosticati disturbi visivi? *

Contrassegna solo un ovale.

- Sì
- No
- Preferisco non rispondere

40. Se sì, che tipo di disturbo visivo?

41. Ti sono mai stati diagnosticati disturbi uditivi? *

Contrassegna solo un ovale.

- Sì
- No
- Preferisco non rispondere

42. Se sì, che tipo di disturbo uditivo?

43. Ha mai sperimentato l'esperienza della sinestesia (condizione per la quale quando si subisce uno stimolo, se ne prova un secondo in contemporanea. Ad esempio: si vede un colore quando si sente una musica, si vede una forma se si sente un sapore, …)? *

Questi contenuti non sono creati né avallati da Google.

Google Moduli
