# Supplementary material for: Assessment of the role of emotions in audiovisual associations through an enactive approach
Source: PLoS One. 2025 May 23;20(5):e0322449. doi: 10.1371/journal.pone.0322449 (PMC12101654; doi:10.1371/journal.pone.0322449)
Supplement: S2 File — English translation of the complete questionnaire, providing the same questions as in S1 File for international readers. (PDF) [file pone.0322449.s005.pdf]

ASSISI TEST

* Indica una domanda obbligatoria

1. Email *

Basic Information

What is the study about?

This study has two main objectives:

• In the short term: studying the relationship between music, images and emotion;

• In the long term: develop a multisensorial platform to enable sensorial augmentation

What will I be asked to do?

In the first session you will carry out short tests to check your visual and musical skills,

In the second you will be able to listen to songs, create images based on the music and express the emotions that what you heard provokes you.

In the last test, you will be shown images and asked what emotions they make you feel.

How long does it take?

You will be asked to participate in 3 sessions of approximately 15/20 minutes each. All sessions will take place on a web platform to which you will have access via a link and you will be free to choose when to complete them. During the sessions you will be exposed to intense colors and music that can trigger emotional reactions.

Before starting the test, please complete this form. After a few days you should receive instructions on how to participate to the study.

If you have any question, feel free to email me at costanza.cenerini@unicampus.it.

2. Name and Surname *

____

3. Date of birth *

____

Esempio: 7 gennaio 2019

4. Sex *

Contrassegna solo un ovale.

- Male
- Female
- I'd rather not answer

5. Nationality *

____

6. Are you right or left-handed? *

Contrassegna solo un ovale.

- Right
- Left
- I use both hands indiferently

Musical Background

7. How much time do you usually spend listening to music every day? *

Contrassegna solo un ovale.

- Less than 20 minutes
- 20 minutes - 1 hour
- 1-3 hours
- more than 3 hours

O a scale from 1 to 10 indicate your liking of each musical genre:

8. Classical *

Contrassegna solo un ovale.

1	2	3	4	5	6	7	8	9	10
◯	◯	◯	◯	◯	◯	◯	◯	◯	◯

9. Pop *

Contrassegna solo un ovale.

1	2	3	4	5	6	7	8	9	10
◯	◯	◯	◯	◯	◯	◯	◯	◯	◯

10. Rock *

Contrassegna solo un ovale.

1	2	3	4	5	6	7	8	9	10
◯	◯	◯	◯	◯	◯	◯	◯	◯	◯

11. Jazz and Blues *

Contrassegna solo un ovale.

1	2	3	4	5	6	7	8	9	10
◯	◯	◯	◯	◯	◯	◯	◯	◯	◯

12. Hip hop and rap *

Contrassegna solo un ovale.

1	2	3	4	5	6	7	8	9	10
◯	◯	◯	◯	◯	◯	◯	◯	◯	◯

13. Country-Western *

Contrassegna solo un ovale.

1	2	3	4	5	6	7	8	9	10
◯	◯	◯	◯	◯	◯	◯	◯	◯	◯

14. Electronic *

Contrassegna solo un ovale.

1	2	3	4	5	6	7	8	9	10
◯	◯	◯	◯	◯	◯	◯	◯	◯	◯

15. Soundtrack *

Contrassegna solo un ovale.

1	2	3	4	5	6	7	8	9	10
◯	◯	◯	◯	◯	◯	◯	◯	◯	◯

16. Have you ever taken part to a music lesson? *

Contrassegna solo un ovale.

- Yes
- No *Passa alla domanda 20.*

Musical Background

17. How long have you studied for? *

Contrassegna solo un ovale.

	0	1	2	3	4	5	6	7	8	9	10	
Less												For 10 or more years

18. Which musical instruments have you studied? *

____

19. When was the last time you took a class? *

Contrassegna solo un ovale.

- Less than a year ago
- Less than 2 years ago
- Less than 5 years ago
- Less than 10 years ago
- More than 10 years ago

Artistic background

On a scale from 1 to 10, indicate how emotional you find these colours and shapes:

20. Red *

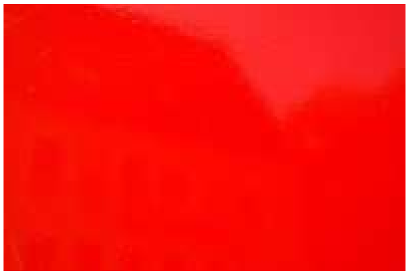

Contrassegna solo un ovale.

1	2	3	4	5	6	7	8	9	10
◯	◯	◯	◯	◯	◯	◯	◯	◯	◯

21. Yellow *

Contrassegna solo un ovale.

1	2	3	4	5	6	7	8	9	10
◯	◯	◯	◯	◯	◯	◯	◯	◯	◯

22. Blue *

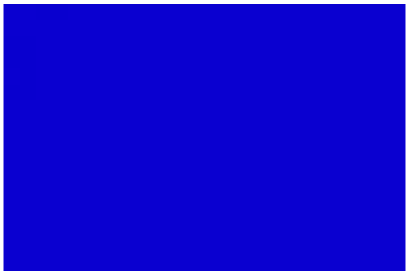

Contrassegna solo un ovale.

1	2	3	4	5	6	7	8	9	10
◯	◯	◯	◯	◯	◯	◯	◯	◯	◯

23. Orange *

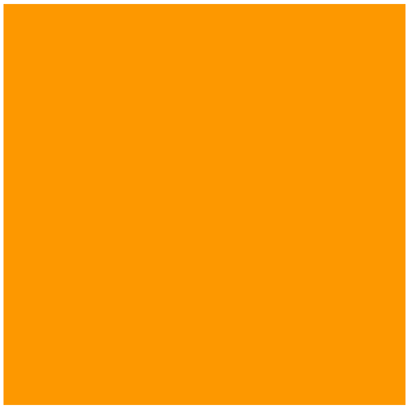

Contrassegna solo un ovale.

1	2	3	4	5	6	7	8	9	10
◯	◯	◯	◯	◯	◯	◯	◯	◯	◯

24. Green *

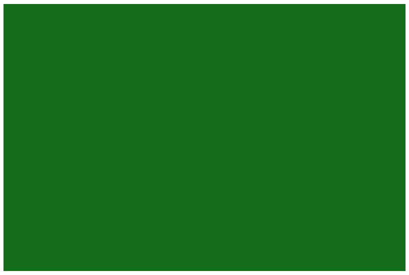

Contrassegna solo un ovale.

1	2	3	4	5	6	7	8	9	10
◯	◯	◯	◯	◯	◯	◯	◯	◯	◯

25. Purple *

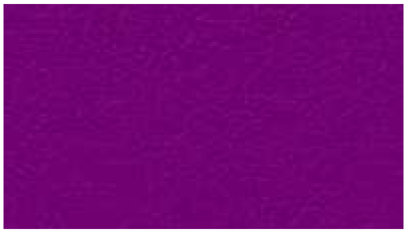

Contrassegna solo un ovale.

1	2	3	4	5	6	7	8	9	10
◯	◯	◯	◯	◯	◯	◯	◯	◯	◯

26. White *

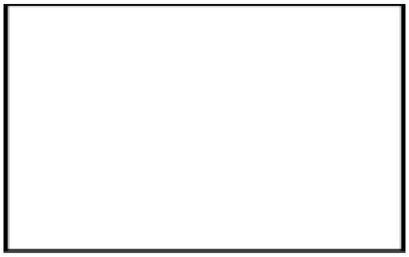

Contrassegna solo un ovale.

1	2	3	4	5	6	7	8	9	10

27. Back *

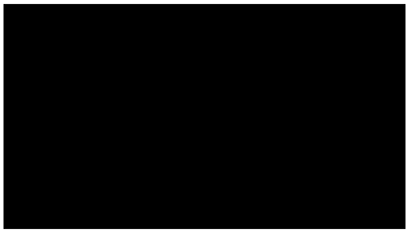

Contrassegna solo un ovale.

1	2	3	4	5	6	7	8	9	10

28. Grey *

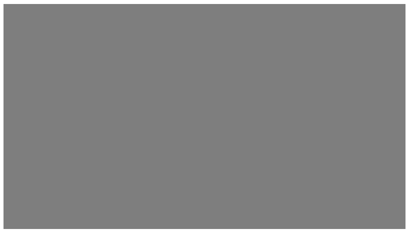

Contrassegna solo un ovale.

1	2	3	4	5	6	7	8	9	10

29. Hexagon *

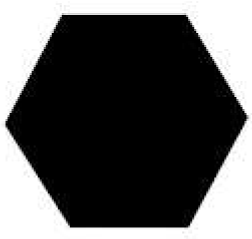

Contrassegna solo un ovale.

1	2	3	4	5	6	7	8	9	10

30. Triangle *

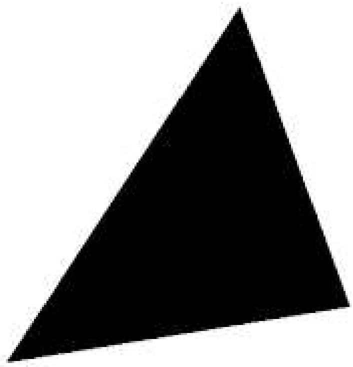

Contrassegna solo un ovale.

1	2	3	4	5	6	7	8	9	10

31. Square *

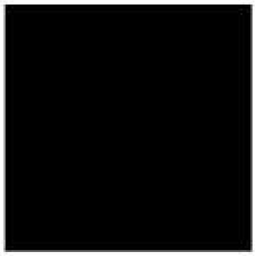

Contrassegna solo un ovale.

1	2	3	4	5	6	7	8	9	10

32. Ellipse *

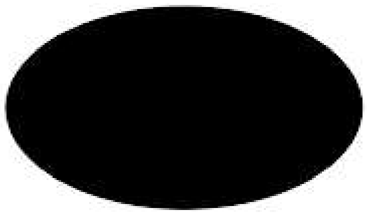

Contrassegna solo un ovale.

1	2	3	4	5	6	7	8	9	10
◯	◯	◯	◯	◯	◯	◯	◯	◯	◯

33. Circle *

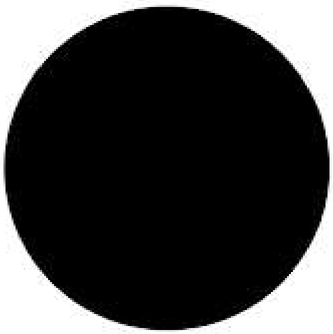

Contrassegna solo un ovale.

1	2	3	4	5	6	7	8	9	10
◯	◯	◯	◯	◯	◯	◯	◯	◯	◯

34. Splash *

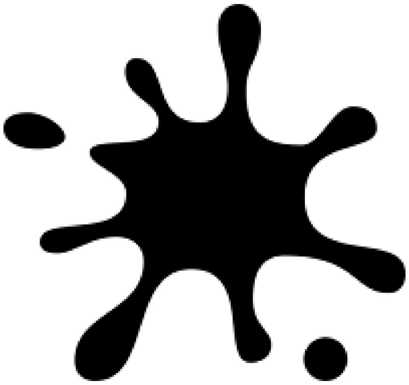

Contrassegna solo un ovale.

1	2	3	4	5	6	7	8	9	10
◯	◯	◯	◯	◯	◯	◯	◯	◯	◯

35. Have you ever taken any lesson in art history, painting, drawing, sculpting or similar? *

Contrassegna solo un ovale.

◯ Yes

◯ No *Passa alla domanda 39.*

Artistic Background

36. What type of lessons have you taken part to? *

____

37. For how long? *

Contrassegna solo un ovale.

	0	1	2	3	4	5	6	7	8	9	10	
Less	◯	◯	◯	◯	◯	◯	◯	◯	◯	◯	◯	More than a year

38. When did you take your last lesson? *

Contrassegna solo un ovale.

- ◯ Less than a year ago
- ◯ Less than 2 years ago
- ◯ Less than 3 years ago
- ◯ Less than 10 years ago
- ◯ More than 10 years ago

Medical Background

39. Were you ever diagnosed with visual impairment? *

Contrassegna solo un ovale.

- ◯ Yes
- ◯ No
- ◯ I'd rather not say

40. If you were, what kind of impairment was it?

41. Were you ever diagnosed with hearing impairment? *

Contrassegna solo un ovale.

- Yes
- No
- I'd rather not say

42. If you were, what kind of impairment was it?

43. Have you ever experienced synesthesia (a condition whereby when you experience one stimulus, you experience a second at the same time. For example: you see a colour when you hear music, you see a shape when you hear a taste, ...)? If you have, could you describe it? *

Questi contenuti non sono creati né avallati da Google.

Google Moduli
